# Improving Communication of Discharge Medications to General Practitioners from Inpatient Psychiatric Wards: A Quality Improvement Project

**DOI:** 10.1192/bjo.2025.10434

**Published:** 2025-06-20

**Authors:** Dylan Thiarya, Mizanoor Rahman, Risha Ruparelia

**Affiliations:** North London NHS Foundation Trust, London, United Kingdom

## Abstract

**Aims:** Upon discharge from an inpatient psychiatric unit, effective communication regarding discharge medications with general practitioners (GPs) is vital for continuity of care. Delaying the transfer of this information may compromise patient safety. According to their guidance, The Royal College of Psychiatrists expect discharge summaries to be sent within 7 days.

The aim of this quality improvement project (QIP) is to improve the time taken for GPs to receive discharge medication information to 7 days, achieving a rate of 100% over 9 months.

**Methods:** Baseline data was collected for patients discharged in May and June 2024 from two acute psychiatric wards at Edgware Community Hospital. Outcome measures included completion of a discharge summary and the time taken for it to be sent to the GP.

For the first PDSA cycle, a discharge notification form containing only vital information for GPs, and therefore a more succinct method of communication, was created. This form consisted of patient demographics and discharge medication, and was implemented for doctors to complete within a 24-hour period. Post-intervention, in addition to previous outcome measures, completion of a discharge notification and the time taken for it to be sent to the GP was also reviewed.

The second PDSA cycle intervention involved meeting with senior administrative colleagues to address ward clerk cover, and educating new doctors on the discharge notification template.

**Results:** Baseline data showed that a total of 57 patients were discharged; 3 were excluded as they were transferred elsewhere. Of the 54 discharge summaries, 30% were not sent within 7 days to the GP.

After the introduction of the discharge notification, 51% of patients had discharge summaries delayed beyond 7 days and of these around 50% were due to administrative issues, 5% had a discharge notification sent within 24 hours and 13% had a notification sent within 7 days of discharge.

After the second intervention, 30% of discharge summaries were delayed beyond 7 days due to doctors completing them late. However, 100% of these had discharge notifications sent within 24 hours.

**Conclusion:** This QIP emphasised the importance of communication with administrative and new medical staff. It highlighted the discharge notification’s role as a safeguard when there is a delay in discharge summary completion. This simple intervention could be replicated across other inpatient units to ensure continuity of care in the community.

